# Newer insights into the role of miRNA a tiny genetic tool in psychiatric disorders: focus on post-traumatic stress disorder

**DOI:** 10.1038/tp.2016.220

**Published:** 2016-11-15

**Authors:** V V Giridharan, R A Thandavarayan, G R Fries, C Walss-Bass, T Barichello, N J Justice, M K Reddy, J Quevedo

**Affiliations:** 1 Translational Psychiatry Program, Department of Psychiatry and Behavioral Sciences, McGovern Medical School, The University of Texas Health Science Center at Houston (UTHealth), Houston, TX, USA; 2 Department of Cardiovascular Sciences, Centre for Cardiovascular Regeneration, Houston Methodist Research Institute, Houston, TX, USA; 3 Center of Excellence on Mood Disorders, Department of Psychiatry and Behavioral Sciences, McGovern Medical School, The University of Texas Health Science Center at Houston (UTHealth), Houston, TX, USA; 4 Neuroscience Graduate Program, The University of Texas Graduate School of Biomedical Sciences at Houston, Houston, TX, USA; 5 Center for Metabolic and Degenerative Diseases, Institute of Molecular Medicine, The University of Texas Health Sciences Center, Houston, TX, USA; 6 Clinical and Translational Research Program on Traumatic Stress, Department of Psychiatry and Behavioral Sciences, Mc Govern Medical School, Houston, TX, USA; 7 Department of Psychiatry and Human Behavior, Warren Alpert Medical School of Brown University, Providence, RI, USA; 8 Laboratory of Neurosciences, Graduate Program in Health Sciences, Health Sciences Unit, University of Southern Santa Catarina (UNESC), Criciúma, Brazil

## Abstract

Post-traumatic stress disorder (PTSD) is a mental disorder occurring in about 2–9% of individuals after their exposure to life-threatening events, such as severe accidents, sexual abuse, combat or a natural catastrophe. Because PTSD patients are exposed to trauma, it is likely that epigenetic modifications have an important role in disease development and prognosis. For the past two decades, abnormal expression of the epigenetic regulators microRNAs (miRs) and miR-mediated gene regulation have been given importance in a variety of human diseases, such as cancer, heart disease and viral infection. Emerging evidence supports a role for miR dysregulation in psychiatric and neurological disorders, including schizophrenia, bipolar disorder, anxiety, major depressive disorder, autism spectrum disorder and Tourette's syndrome. Recently mounting of evidence supports the role of miR both in preclinical and clinical settings of psychiatric disorders. Abnormalities in miR expression can fine-tune the expression of multiple genes within a biological network, suggesting that miR dysregulation may underlie many of the molecular changes observed in PTSD pathogenesis. This provides strong evidence that miR not only has a critical role in PTSD pathogenesis, but can also open up new avenues for the development of diagnostic tools and therapeutic targets for the PTSD phenotype. In this review, we revisit some of the recent evidence associated with miR and PTSD in preclinical and clinical settings. We also discuss the possible clinical applications and future use of miRs in PTSD therapy.

## Introduction

Post-traumatic stress disorder (PTSD) is a mental illness that results from the experience or witnessing of traumatic or life-threatening events. It was first officially recognized in the *Diagnostic and Statistical Manual of Mental Disorders*, 3rd ed. (DSM III) in 1980, and since then knowledge has accumulated about the characteristics, symptomatology and epidemiology of PTSD as well as assessment and treatment of individuals suffering from this disorder. According to DSM-5, the diagnostic criteria for PTSD includes experiencing at least one symptom from the re-experiencing factor, at least one symptom from the avoidance factor, two or more symptoms from the negative alterations in cognitions and mood factor, and two or more symptoms from the alterations in arousal and reactivity factor, along with significant functional impairment and at least a one-month duration of the symptoms.^1^ It can be categorized under two types, acute or chronic. In acute PTSD, symptoms last for at least 1 month but <3 months after the traumatic event. In chronic PTSD, symptoms last for more than 3 months after exposure to trauma.^2^ PTSD patients with either type frequently show socio-behavioral problems and have a higher risk of alcohol and drug abuse. In addition, structural changes in the prefrontal cortex, amygdala and hippocampus, and biochemical changes such as increase in norepinephrine/cortisol in urine, elevated total cholesterol in the blood and higher levels of norepinephrine in cerebrospinal fluid (CSF) are hallmarks of PTSD.^3, 4, 5, 6^ PTSD included as a new chapter in DSM-5 on trauma and stressor-related disorders. This move from DSM-IV, which addressed PTSD as an anxiety disorder, and its symptoms, includes depression, outbursts of anger, self-destructive behavior, and feelings of shame, self-blame and distrust.^7^ Notable biological findings in PTSD include derangements in the noradrenergic/sympathetic brain systems and the hypothalamic–pituitary–adrenal axis, increased CSF concentrations of corticotropin-releasing factor; reduced volume of the hippocampus, functional differences in responding of fear system brain regions, such as hyperactivation of the amygdala and hypoactivation of the prefrontal cortex; sleep disturbances, measures of hyperarousal in response to stimuli and of delayed habituation to loud noises, and evidence for impaired conditioned fear extinction recall.^8^ The currently available pharmacotherapies target the different phenotypes of PTSD, such as anxious, depressive, externalizing and dissociative. Multisite randomized clinical trials have noted the efficacy of FDA-approved selective serotonin reuptake inhibitors and serotonin–norepinephrine reuptake inhibitors for PTSD treatment. Although alternatives to serotonin reuptake inhibitors and mixed serotonin–norepinephrine reuptake inhibitors in PTSD would be desirable, little data supports the use of other antidepressants, benzodiazepines, atypical antipsychotics for clinical symptoms. The comorbid psychotic features such as nightmares and sleep disturbance in PTSD treated by Prazosin and alpha-1-adrenergic antagonist that attenuates noradrenergic-mediated suppression of rapid eye movement sleep in preclinical work. However, there are currently no pharmacotherapy treatments that can be recommended clinically for the prevention of PTSD development post-trauma.^8^ The overall response rate of serotonin reuptake inhibitors treatment is about 60% in PTSD patients and only 20–30% of patients achieve complete remission. Unfortunately, there have been no other new medications approved to treat PTSD in the past 10 years.^9^

By definition PTSD requires exposure to a traumatic event. As genes are sensitive to stress and trauma, epigenetic alterations have received attention as a possible mechanism for the development and persistence of PTSD. miRNAs (miRs) can be considered important players in the epigenetic control of gene expression that not only have its role in regulation of many cellular and developmental processes but also can be developed as a novel diagnostic tool or target for therapeutic intervention.^10, 11^ Over the past 15 years, many epigenetic markings such as DNA methylation, post-translational histone modifications and histone variants have moved center attention in many areas of translational and experimental medicine, including neurology and psychiatry.^12^ In addition, the epigenetic regulation of catechol-o-methyltransferase (COMT), an enzyme, which degrades dopamine, is associated with the impaired fear inhibition in PTSD.^13^ Besides DNA methylation and histone modifications, recently non-coding RNAs (ncRNAs) have gained considerable attention in diverse fields. It is demonstrated that RNAs are not only an intermediate molecule between DNA and protein. Some RNAs that are not translated into protein can act as functional molecules known as ncRNAs and can regulate normal cellular function and gene expression. The principle ncRNA molecules that participate in gene expression, such as ribosomal RNA and transfer RNA were discovered in the early 1950s. Other ncRNAs such as small nuclear RNAs, small interfering small cajal body specific RNAs, small nucleolar RNAs, long non-coding RNAs, long intergenic non-coding RNAs, piwi-interacting RNAs and circular RNAs were more recently discovered and have been shown to have a remarkable variety of biological functions. In 1993, the revolution in RNA biology started by the discovery of the first miR, in the nematode *Caenorhabditis elegans*, Lin-4 by the efforts of Ambros's and Ruvkun's laboratories.^14, 15^ Seven years later the second miR, let-7a heterochronic gene of *C. elegans* was reported by Reinhart *et al.*^16^ at Ruvkun's laboratory. Currently, thousands of miRs have been identified in humans and other species. In eukaryotic organisms, the discovery of miRs was a huge revolution because it depicted their importance in post-transcriptional events.^17^ Recently, accumulating evidences explores the role of miR in psychiatric diseases such as schizophrenia, autism and bipolar disorders. In this review, we aim to brief the recent highlights in the neurobiology of miR associated with PTSD and its comorbid depression, and examine the support for their involvement in psychiatric pathophysiology. We also addressed the possible clinical applications for miR in PTSD treatment and what is needed for the field to progress.

## Biogenesis and functions of miR

**Figure 1:**
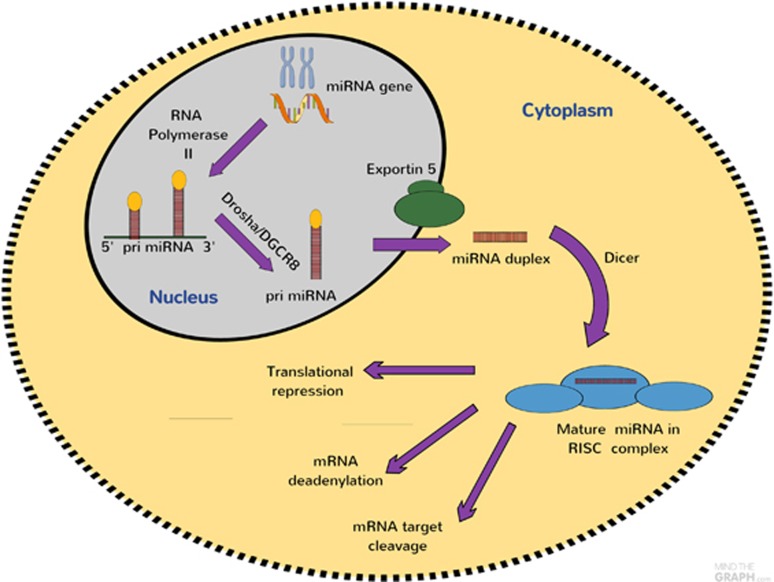
microRNAs (miRs) biogenesis and function. miRs are transcribed by RNA polymerase II or III as pri-miR, and are processed in the nucleus by Drosha into pre-miRs. The pre-miR, is exported to the cytoplasm by exportin-5 and further cleaved in a complex composed of Dicer and trans-activation response RNA-binding protein. The functional strand of mature miR is incorporated into the RNA-induced silencing complex (RISC). As a part of this complex, the mature miR regulates gene expression by binding to partially complementary sequences in the 3′-untranslated regions (3′UTRs) of target mRNAs, leading to transcriptional repression and transcriptional activation.

miRs are genomically-encoded, single stranded, non-coding RNA molecules with 19–24 nucleotides that anneal with complementary sequences to messenger RNA (mRNA), thereby regulating protein expression. miR reduce the transcription and translation of mRNA, thereby down-regulating gene expression.^15^ miR are transcribed by RNA polymerase II as long primary transcripts characterized by hairpin structures called pri-miR, and are processed in the nucleus by RNA polymerase III Drosha into 70–100 nucleotide long precursor miRs in combination with cofactors such as DGCR8. The product of pri-miR cleavage is exported to the cytoplasm by exportin-5, a member of the Ran-dependent nuclear transport receptor family and further cleaved in a complex composed of RNase III Dicer and the trans activating response RNA-binding into a miR duplex.^18^ Dicer and several other RNA-binding proteins, such as Ago2, protein activator of PKR and trans-activation response RNA-binding protein, incorporate one strand of mature miR duplexes into the ribosome-induced silencing complex. The miR-associated ribosome-induced silencing complex binds to the target miR to inhibit its translation or cause the degradation of the target miR. miRs silence their target mRNAs by a variety of mechanisms at the post-transcriptional level by binding to the 3′-untranslated regions (3′UTRs) or the open reading frames of target genes, leading to the degradation of target mRNAs or repression of mRNA translation (Figure 1). miR was originally considered to have no biological function and to be degraded; however, recent evidence suggests that it can be used as a functional strand and may have significant biological roles. It is believed that there are as many 1000 miR in the human genome and that up to 30% of human genes are regulated by miR. For some human genes, more than one miR may be involved in their regulation and more than 80% of conserved miR is tissue-specific.

## miR signatures in psychiatric disorders

**Figure 2:**
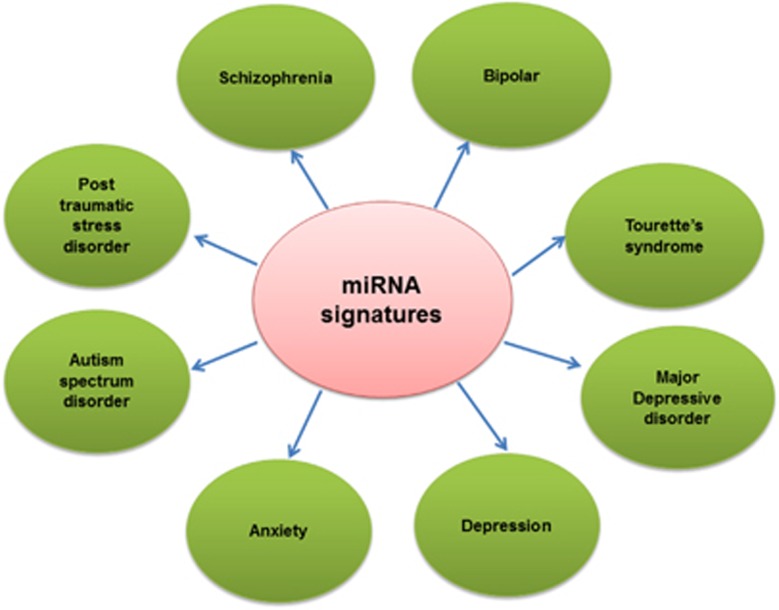
MicroRNA in various psychiatric disorders.

The common etiology of psychiatric disorders includes (1) genetic factors such as polymorphisms, gene deletions or insertions, gene amplification and gene translocation (2) environmental factors such as stress. The combination of genetic and environmental factors is known to lead the development of most psychiatric disorders.^19^ Environmental factors by means of regulating epigenetic mechanisms can interact with the genome to have long-term consequences for brain plasticity and behavior. These mechanisms include histone modification, DNA methylation and post-transcriptional regulation by ncRNA such as miRs. In psychiatry, we are now in the first era of miR biology approach. Recent evidence in psychiatric disorders such as schizophrenia and bipolar disorder supports a role for miRs as possible mediators of the susceptibility, onset, diagnosis and treatment of these disorders.^20, 21^ A recent study by Hauberg *et al.* provides evidence for the role of miR in the etiology of schizophrenia. Schizophrenia risk genes were more likely to be regulated by miRs, as revealed by gene set analyses with the strongest enrichment for targets of miR-9-5p, miR-485-5p and miR-137.^22^ miR was also suggested as a biomarker for schizophrenia in CSF and peripheral blood. Mononuclear leukocyte-based miR profiling identified seven miRs, that is, miR-34a, miR- 449a, miR-564, miR-432, miR-548d, miR-572, miR-652 and had a high discriminating accuracy for schizophrenia.^23^ In addition, a large cluster of 17 miRs on chromosome 14q32 were reported to have the potential to serve as biomarkers for schizophrenia.^24^ In the same vein, miR-499, miR-708 and miR-1908 were shown to have significant association with bipolar disorder.^25^ In plasma from drug-free manic patients, miR-134 was shown to be downregulated compared with controls.^26^ The recent study suggests that a set of 13 differentially expressed serum miRs might serve as a possible non-invasive biomarker for autism spectrum disorder.^27^ Tourette syndrome (TS) is a childhood neuropsychiatric disorder characterized by multiple motor and one or more vocal tics. The syndrome is commonly associated to comorbid conditions such as attention deficit hyperactivity disorder and obsessive compulsive disorder. Network and gene-ontology analysis revealed that miR-429 is significantly under expressed in TS patients with respect to controls.^28^ The miR signatures in different psychiatric disorders showed in Figure 2. Despite this earlier promising work, there have been only a handful of preclinical and clinical studies investigating the role of miRs in other psychiatric disorders such as PTSD.

## Implications of miR in PTSD

By definition PTSD requires exposure to a traumatic event. As genes are sensitive to stress and trauma, epigenetic alterations have received attention as a possible mechanism for the development and persistence of PTSD. Epigenetic modifications, including DNA methylation, histone modifications and ncRNAs, have been implicated in a number of complex diseases such as cardiovascular disease, cancer and neurological diseases.^29^ In regards to PTSD, the recently identified allele specific DNA methylation of *FKBP5*, a potential candidate gene for PTSD, has been suggested to mediate gene-childhood trauma interactions.^30^ Table 1, summarizes reports of dysregulated miR in both preclinical and clinical models of PTSD. There is now evidence that miRs are involved in brain development and disease and in control of neurotransmitter release. For example, miR-130a and miR-206 inhibit the synthesis of substance P, whereas interleukin-1α reduces the expression of these miRs, relieving their inhibition.^31^ Abelson *et al.* identified Slit- and Trk-like family member 1 (SLITRK1), a leucine-rich transmembrane protein as involved in TS, a brain disorder potentially induced by miR dysregulation. They subsequently identified two independent pro-bands with a 3′-UTR mutation in a miR-189 binding site.^32^

Depression is a condition that is highly comorbid with PTSD. In the Grady Trauma Project (GTP), the prevalence of PTSD and depression was 28.4% among 6863 at risk participants with high rates of trauma exposure. Also, in 9/11 World Trade Center Health Registry enrollees, 10.1% had PTSD and depression about 10 years after the September 9/11 disaster. Hence it is important to assess the role of miR in both stress and depression and we have tabulated this in Table 2.

## Evidence of miR in preclinical model of PTSD

### miR-1971 as a target gene in PTSD pathobiology

In a study published by Schmidth *et al.*,^66^ the rodent model of electric foot shock was used to induce the PTSD phenotype. The antidepressant drug fluoxetine (effective both in PTSD patients and mice suffering from a PTSD-like syndrome) was administered at a dose of 20 mg kg^−1^ day^−1^. miR profiles in prefrontal cortices dissected from either fluoxetine or control-treated wildtype C57BL/6N mice 74 days after their subjection to either a single traumatic electric footstock or mock-treatment were performed using micro array profiling. The relative expression levels of all potential miR target sequences of miRBase 18.0 by pairwise comparison of the prefrontal cortices miR profiles resulted in an identification of five miR candidate molecules. Validation of these miR candidates by RT-qPCR reaction revealed that the therapeutic action of fluoxetine in shocked mice is associated with a significant reduction in mmu-miR-1971. To our knowledge, this is the first study demonstrating miR expression profiles in a PTSD mouse model. Thus the antidepressant fluoxetine in shocked mice is correlated with significant reduction in prefrontal cortical mmu-miR-1971.^66^

### miR as a potential biomarker in rat model of PTSD

In this study by Balakathiresan *et al.*,^36^ the rat model of learned helplessness stress was used to identify significantly modulated miRs in serum and amygdala after induction of the PTSD phenotype. The stress protocol consisted of a 2 h per day session of immobilization along with tail shocks for three consecutive days. During restraints animals were exposed to 40 electric shocks at varying intervals of 150–210 s. This study identified the significantly modulated miRs in serum after traumatic stress to develop a novel non-invasive PTSD diagnostic biomarker and validated their expression on amygdala based on, it is involvement in regulating fear response under stress.^67^ Accordingly differentially expressed and statistically significant miRs in serum were validated for their presence in amygdala of corresponding animals. The nine stress-responsive miRs, miR-142-5p, miR-19b, miR-1928, miR-223-3p, miR-322^∗^, miR-324, miR-421-3p and miR-463^∗^ identified may have potential as biomarkers for PTSD. Bioinformatics and system biology validation indicated that five of the nine miRs, that is, miR-142-5p, miR-19b, miR-1928, miR-223 and miR-421-3p may have a role in the regulation of genes associated with delayed and exaggerated fear.^36^

### miR-29 family members: key regulators involved in PTSD—heart pathology

This study by Van Rooij *et al.*^68^ examined the effects of short-term stress exposure on heart tissue in a PTSD mouse model and identified the family of miRs involved in heart pathology. The PTSD phenotype was induced by the social defeat model, adapting the different strains of subservient mice by pairing with aggressor mice (SJL strain). The study identified several key gene regulators, members of the miR-29 family (miR-29b) that are involved in the wound-healing process.^43^ It is to be noted that the miR-29 family members have been implicated in arrhythmias, myocardial fibrosis and other heart conditions.^68^

### miR-34c in the pathobiology of PTSD

This study by Li *et al.*^38^ identified miR-34c expression in hypothalamus as an important factor involved in susceptibility to PTSD in a rat model. Rats received the repeated inescapable electric foot shock for six consecutive days for the induction of the PTSD phenotype. The study focused on the relationship among levels of corticotrophin releasing factor receptor (CRFR) 1 mRNA, and miR-34c expression in adult stressed rats and suggested that CRFR1 antagonist could target a positive process including increased levels of miR-34c during acute stress reaction and give a new certification that miR-34c might be closely related with vulnerability to PTSD.

## Evidence of miR in clinical model of PTSD

### miR-125a targets cytokine production in combat veterans with PTSD

This study by Zhou *et al.*^33^ evaluated the role of miR in the immunological dysfunction associated with PTSD. The peripheral blood mononuclear cells (PBMC) and various lymphocyte subsets in blood collected from combat veterans with PTSD were analyzed. The numbers of both PBMC and lymphocyte subsets were significantly increased in combat veterans PTSD patients compared with controls. There was an alteration in immune function such as helper T (Th)1 and Th17 cells were increased and regulator T cells decreased, but Th2 cells remained unaltered in PTSD patients. These data correlated with increased plasma levels of cytokines such as interferon-γ and interleukin-17. The severity in PTSD patients as determined by the clinical scores such as PTSD scores, anxiety scores and depression scores. It was found that increase in PBMC counts, Th1 and Th17 cells seen in PTSD patients correlated with clinical scores. High-throughput analysis exhibited significant alterations in miR expression which correlates with immunological changes in combat veterans with PTSD.^33^

### miR-3130-5p and *DICER1* regulates biological mechanism of PTSD&Dep

This study by Wingo *et al.*^35^ identified for the first time the involvement of *DICER1* and miR in the regulation pathway implicated in the biological mechanism of PTSD and cormorbid depression (PTSD&Dep). *DICER1* is an enzyme that cleaves precursor RNA molecules to produce mature miR. It has been found that blood DICER1 expression was significantly reduced in PTSD&Dep patients, and it was associated with increased amygdala activation to fearful stimuli, a neural correlate for PTSD. Further, genome-wide differential expression survey of miRs in blood in PTSD&Dep reveals the miR-3130-5p was significantly reduced in abundance level in the PTSD&Dep cases.^35^

## Future perspectives of miR in psychiatric diseases

With more than hundreds of miRs identified and knowledge of their role in psychiatric disease becoming clearer, there is the prospect over the coming years, to harness miR in psycho-therapeutics. On the other hand, Miravirsen the first miR-based therapeutics for hepatitis C virus infection entered the phase-II clinical trial.^69^ However, to define miRs as a new target for psychiatric therapy would be premature. Because, delivering miR-targeted therapies to the central nervous system poses a considerable challenge, it is important to identify the target gene. Current understanding on bioinformatics has provided useful information, but precise targets must be identified among the different predicted targets. Thus identification of specific target genes would further deepen our understanding of the mechanisms underlying psychiatric disease pathogenesis. Viral and non-viral vectors are being investigated for their potential to deliver miRs to the central nervous system, in addition, intranasal delivery, a non-invasive method that has also been shown some promise in this regard.^70^ The aberrant miRs implicated in disease or therapeutic effects must be further investigated in animal and other preclinical models, instead of extrapolating from an animal model to a clinical setting.^71^ This could involve overexpression or knockdown of a candidate miR in cell lines and measuring the target gene's protein expression levels as well as behavioral phenotype in preclinical settings. Likewise, aberrant miRs expression identified in preclinical settings should be followed up in clinical or postmortem samples. Identifying new rare variants through sequencing as well as genotyping larger cohorts for more common miR disease risk variants should also be explored. Thus, studies correlating miR profiles with clinical outcomes would be helpful in the development of biomarkers and miR-based therapeutics in the future.

## Conclusion

In summary, the given preclinical and clinical evidences suggest the important role of miRs in PTSD pathophysiology and diagnosis. There is a great expectation for the use of miR measures and genetic data as non-invasive biomarkers for the diagnosis, prognosis and therapeutic appraisal of many illnesses. The fact that differential expression levels of peripheral miR have been associated with several disease processes pertaining to brain tissues suggest the potential use of miR as a new generation of biomarkers and opens new avenues for the treatment of neuropsychiatric conditions. Compare with cancer and cardiovascular field, miR implication in psychiatric research at the nascent stage, hence more detailed understanding of the function of miRs in PTSD pathophysiology and clinical condition may improve the treatments and possibly lead to the clinical application of miRs in PTSD diagnosis, treatment and prognosis.

## Figures and Tables

**Table 1:** miR levels in clinical and preclinical PTSD-related studies

*Species*	*Tissue sample*	*Study design*	*Methods for RNA analysis*	*Main findings*
Human	PBMC	30 PTSD vs 42 healthy controls	miR array, PCRs	↑ miR-570, miR- 219-1-3p, miR-637, miR- 668, miR- 519a*, miR-518f* and ↓ miR- 615-5p* and miR-125a and 181c^33^
Human	PBMC	16 PTSD vs 17 healthy controls	qRT-PCR	↓ hsa-miR-193a-5p in PTSD patients^34^
Human	Blood	34 PTSD&depression vs 20 healthy controls	qPCR	↓ miR- 3130-5p in PTSD&depression^35^
Rat	Serum, amygdala	PTSD rat model (immobilization along with tail shocks)	miR array, TaqMan miR assay	↑ miR-322*, miR-324,miR-463*, miR-674*, miR-142-5p, miR-19b, miR-1928, miR-223-3p, miR-421-3p in serum and amygdala^36^
Rat	Amygdala	Fear conditioning	miR array, TaqMan miR assay,	↓ miR-182 in lateral amygdala^37^
Rat	Hypothalamus	Electric foot shock	RT-PCR	↑ miR-34c expression in hypothalamus^38^
Rat	Frontal cortex	Conditioning model	TaqMan miR assay, RT-qPCR	↑ miR-222 in frontal cortex. ↓ miRs 218, 194, 206 and miRs 224, 142, 126, 296 unchanged^39^
Mice	PFC	PTSD mice model (electric shock)	miR array, RT-qPCR	↓ Levels of mmu-miR 1971 in PFC^40^
Mice	Hippocampus	Fear conditioning	Lentiviral vector for miR knockdown, TaqMan miR assay	90 min after FC, ↑ miR 92a. Selective inhibition of miR92 in CA1 neurons leads to up-regulation of 3 miR 92a target genes^41^
Mice	Amygdala	Fear conditioning	miR array, RT-qPCR	30 min after fear-conditioning, miR-34a ↑ in the amygdala^42^
Mice	Sperm, serum, various brain regions	Early life stress (MSUS)-producing behavioral effects across generations	Deep sequencing, RT-qPCR	miR-375-3p, miR-375-5p, miR-200b-3p, ↑ miR-672-5p and miR-466c-5p in sperm, serum, hippocampus, hypothalamus (not cortex) of adult F1 MSUS mice and in serum and hippocampus (but not sperm) of adult F2 MSUS mice^37^
Mice	Heart	Social defeat model	miR array	PTSD-like symptoms were accompanied by heart injury that wasaccompanied by ↓ miR-29b, miR 302a and let-7d levels^43^
Mice	Hippocampus	Fear conditioning	Lentiviral vector for miR knockdown, TaqMan miR assay	miR-132 levels in hippocampus^44^
Mice	Hippocampus	Contextual fear-conditioning		miR-33 regulates the levels of GABA-related proteins^45^
Mice	Amygdala	Social defeat model	miR array	↑ miR-19b in amygdala^46^
Mice	Various brain regions	Fear conditioning	TaqMan miR assay	Rapid and transient of the primary transcript of miR-132 followed by a in mature miR-132^47^
Mice	Hippocampus, cortex	Battery of behavioral tasks, fear-conditioning	miR array, TaqMan analysis	Deletion of Dicer1 gene in forebrain leads to loss of a miR124, miR-132, miR-137, miR138, miR29a, miR29c^48^
Mice	ILPF	Fear conditioning	Lentiviral vector for miR knockdown, miR overexpression	miR-134 expression in ILPFC, whereas miR-128b in extinction training only Knockdown of miR-128b fear-conditioning memory, while overexpressing it^49^

Abbreviations: IFN, interferon; ILPF, infra-limbic prefrontal cortex; miR, microRNA; MSUS, mice model of unpredictable maternal separation combined with unpredictable maternal stress; PBMC, peripheral blood monocytic cells; PFC, prefrontal cortex; PTSD, post-traumatic stress disorder; RT-qPCR, quantitative real-time polymerase chain reaction.

Symbols: ↑ increase, ↓ decrease.

**Table 2:** miR implication in stress and depression results from clinical and preclinical models

*Species*	*Effect/tissue*	*miR*	*Reference*
Human	Blood	has-miR-130b, miR 505, miR-29b-2, miR-26b, miR22, miR26a, miR664, miR-494, miR629, miR106b, miR103, miR-191, miR128, miR502-3p, miR 374b, miR-132, miR30d, miR-500, miR-770-5p, miR-589, miR-183, miR-574-3p, miR-140-3p, miR-335, miR-361-5 phas-let-7g, has-let-7d, has-let-7e, has-miR-34c-5p, has-let-7f	Bocchio-Chiavetto *et al.*^50^
Human	Blood	Has- miR-107, miR-133a, miR-148a, miR-200c, miR381, miR-425-3p,miR-494, miR-517b, miR-579, miR-589, miR-636, miR-652, miR-941, miR-1243	Belzeaux *et al.*^51^
Human	Plasma	miR-144-5p	Wang *et al.*^52^
Human	Blood	miR-34b-5p and miR-34c-5p	Sun *et al.*^53^
Human	Blood	miR-182, miR-132 and miR-182	Li *et al.*^54^
Human	Blood	miR-26b, miR-1972, miR-4485, miR-4498 and miR-4743	Fan HM *et al.*^55^
Human	Blood/Brain	miR-135	Issler *et al.*^56^
Human	Blood	miR-320, miR-451, miR-17-5p, miR-223-3p	Camkurt *et al.*^57^
Human	Post-mortem prefrontal cortex brain tissue/ depressant patient blood	miR-1202	Lopez *et al.*^58^
Human/postmortem brain studies	Prefrontal cortex	has- miR-10a, miR-20a, miR-20b, miR-27a, miR-33a,miR-137, miR-142-3p, miR-142-5p, miR-148b, miR-155, miR-190miR-376a	Smalheiser *et al.*^59^
Human	Blood and brain	miR135	Issler *et al.*^56^
Rat	Serum	miR-16	Zurawek *et al.*^60^
Rat	Immobilization stress/ hippocampus CA1, amygdala	miR-132, miR-134, miR-183, let-7a-a, miT-9-1, miR-124a-1	Meerson *et al.*^61^
Rat	Unpredictable chronic mild stress/hippocampus	miR-125a, miR-298, miR-130b, miR-135a, miR-323, miR-503, miR-15b, miR-532 miR-7a, miR-212, miR-124, miR-139, miR-182	Cao *et al.*^34^
Rat	Inescapable shock/frontal cortex	mmu-miR-184, Mmu-miR-197, mmu-miR-107, mmu-miR-329, mmu-miR-125a-5p, mmu-miR-872, mmu-miR-181c, mmu-miR-18a, mmu-miR-29b, mmu-let-7a. Rno-let-7e, rno-miR-20a	Smalheiser *et al.*^62^
Mice	Unpredictable mild stress/frontal lobe and hippocampus	miR-132, miR-18a, miR-134, miR-124a miR-18a	Pan and Liu^63^
Mice	Restraint stress/ frontal cortex	miR-9, miR26a/b, miR-29b,miR-30b, miR-30b/c, miR-30c, miR-30e, miR125a, miR-126-3p,miR-129-3p, miR-207, miR-212, miR351, miR423, miR-487b, miR-494, miR-690, miR-691, miR-709, miR711 and let-7a-e let-7	Rinaldi *et al.*^64^
Mice	Stressed/hypothalamus	miR-18, miR-12a	Shimizu *et al.*^65^

Abbreviation: miR, microRNA.
